# VEGF to CITED2 ratio predicts the collateral circulation of acute ischemic stroke

**DOI:** 10.3389/fneur.2022.1000992

**Published:** 2022-09-30

**Authors:** Minyi Lu, Yuben Liu, Zhiqiang Xian, Xiaoyao Yu, Jian Chen, Sheng Tan, Peidong Zhang, Yang Guo

**Affiliations:** ^1^Department of Neurology, Zhujiang Hospital, Southern Medical University, Guangzhou, China; ^2^Department of Cardiology, Heart Center, Zhujiang Hospital, Southern Medical University, Guangzhou, China

**Keywords:** collateral circulation, VEGF/CITED2, AIS, PBMCs, biomarker

## Abstract

**Objective:**

The research objective was to evaluate the predicting role of the vascular endothelial growth factor to CBP/P300-interacting transactivator with Glu/Asp-rich C-terminal domain 2 Ratio (VEGF/CITED2) from peripheral blood mononuclear cells (PBMCs) in the collateral circulation of acute ischemic stroke (AIS).

**Methods:**

In an observational study of patients with AIS, the western blot was applied to test the protein expression of VEGF and CITED2. Then, we calculated the VEGF/CITED2 and collected other clinical data. Binary logistic regression analysis between collateral circulation and clinical data was performed. Finally, receiver operating characteristic (ROC) curve analysis was used to explore the predictive value of VEGF/CITED2.

**Results:**

A total of 67 patients with AIS were included in the study. Binary logistic regression analysis indicated the VEGF/CITED2 (OR 165.79, 95%CI 7.25–3,791.54, *P* = 0.001) was an independent protective factor. The ROC analyses showed an area under the ROC curve of the VEGF/CITED2 was 0.861 (95%CI 0.761–0.961). The optimal cutoff value of 1.013 for VEGF/CITED2 had a sensitivity of 89.1% and a specificity of 85.7%.

**Conclusion:**

In patients with AIS, the VEGF/CITED2 was related to the establishment of collateral circulation. The VEGF/CITED2 is a potentially valuable biomarker for predicting collateral circulation.

**Clinical trial registration:**

ClinicalTrials.gov, identifier: NCT05345366.

## Introduction

Acute ischemic stroke (AIS) is a common neurological event of disability and death (1). Its clinical prognosis varies greatly, such as complete recovery, neurological deficit, and death. This diversity is mainly accounted for by collateral circulation (2). When a cerebral vessel develops stenosis or occlusion, the blood can reach the surrounding peri-infarct zone (referred to as the penumbra) through collateral circulation so that the local ischemic brain tissue can be saved (3). Thus, collateral circulation has a significant impact on the recovery of neurological function and clinical prognosis (4).

Imaging examination is the major method to evaluate collateral circulation, such as transcranial Doppler, traditional single-phase CT angiography (CTA), MR angiography, and digital subtraction angiography (DSA), which depends on specialty devices and professional technicians (5). At present, there is still a lack of an effective and sensitive biomarker to predict collateral circulation. Therefore, the search for the biomarker is beneficial for predicting collateral circulation and judging clinical prognosis.

At present, the establishment of collateral circulation is a hot topic. Ischemic brain tissues recruit peripheral blood mononuclear cells (PBMCs) during the establishment of collateral circulation, among which PBMCs play a significant role by secreting vascular endothelial growth factor (VEGF) (6–8). Multiple studies have observed the high expression of VEGF can promote vasculature remodeling, promote the formation of angiogenesis, and reduce brain infarct volume in the middle cerebral artery occlusion rat model (9, 10). Of those, the most widely studied pathway is the hypoxia-inducible factor-1α (HIF-1α)—VEGF pathway.

Bhattacharya et al. found that CBP/P300-interacting transactivator with Glu/Asp-rich C-terminal domain 2 (CITED2) acts as a molecular switch in the HIF-1α-VEGF pathway (11). CITED2 is a nuclear protein widely expressed in mammalian cells and plays a significant role in the development and growth of cells (12). CITED2 is mainly expressed on peripheral blood mononuclear cells (PBMCs) (13). Under hypoxic conditions, CITED2 involves in the HIF-1α-mediated VEGF angiogenesis pathway (11). In addition, CITED2 may activate the peroxisome proliferator-activated receptor (PPAR) pathway which inhibits VEGF expression (14). However, the mechanism of the action of CITED2 is yet to be elucidated in AIS. Thus, we suggest that CITED2 regulates the VEGF-mediated angiogenesis pathway. The VEGF/CITED2 was better at predicting collateral circulation due to the combined effect of VEGF and CITED2. However, there is no direct evidence that the VEGF/CITED2 in PBMCs can predict the collateral circulation of AIS.

Alberta Stroke Program Early CT Score (ASPECTS), a semiquantitative approach in non-contrast CT, was originally developed to evaluate infarct size, clinical prognosis, and the probability of hemorrhagic transformation in patients with AIS (15). Because of the close relationship between clinical prognosis and collateral circulation, ASPECTS can also be used as an indirect score for evaluating collateral circulation (16). With the imaging methods developing, ASPECTS is implemented into diffusion-weighted imaging (DWI) to evaluate the collateral circulation. Furthermore, DWI is indicated with ischemic tissue of early AIS. Thus, as compared to ASPECTS, DWI-ASPECTS more clearly reflects the collateral circulation of AIS. Yuan et al. evaluated the predictive value of DWI-ASPECTS and conducted a receiver operating characteristic (ROC) curve analysis in 178 patients with AIS. They found the area under the ROC curve (AUC) of 0.932, the sensitivity of 81%, and a specificity of 94.1% (17). In contrast to DSA, CTA, CTP, and perfusion-weighted imaging (PWI), DWI-ASPECTS offers the advantages of being non-invasive and does not injection of contrast agents. At the same time, it has a few disadvantages, such as time-consuming and patient cooperation. According to practical conditions at our hospital, most patients with AIS were examined by DWI. Only a few patients were examined by DSA, CTA, CTP, and PWI. Therefore, we explored the relationship between VEGF/CITED2 and collateral circulation by DWI-ASPECTS. In this study, it provides an objective basis for VEGF/CITED2, as an effective biomarker, to predict the collateral circulation of AIS.

## Methods

### Study design

**Figure 1:**
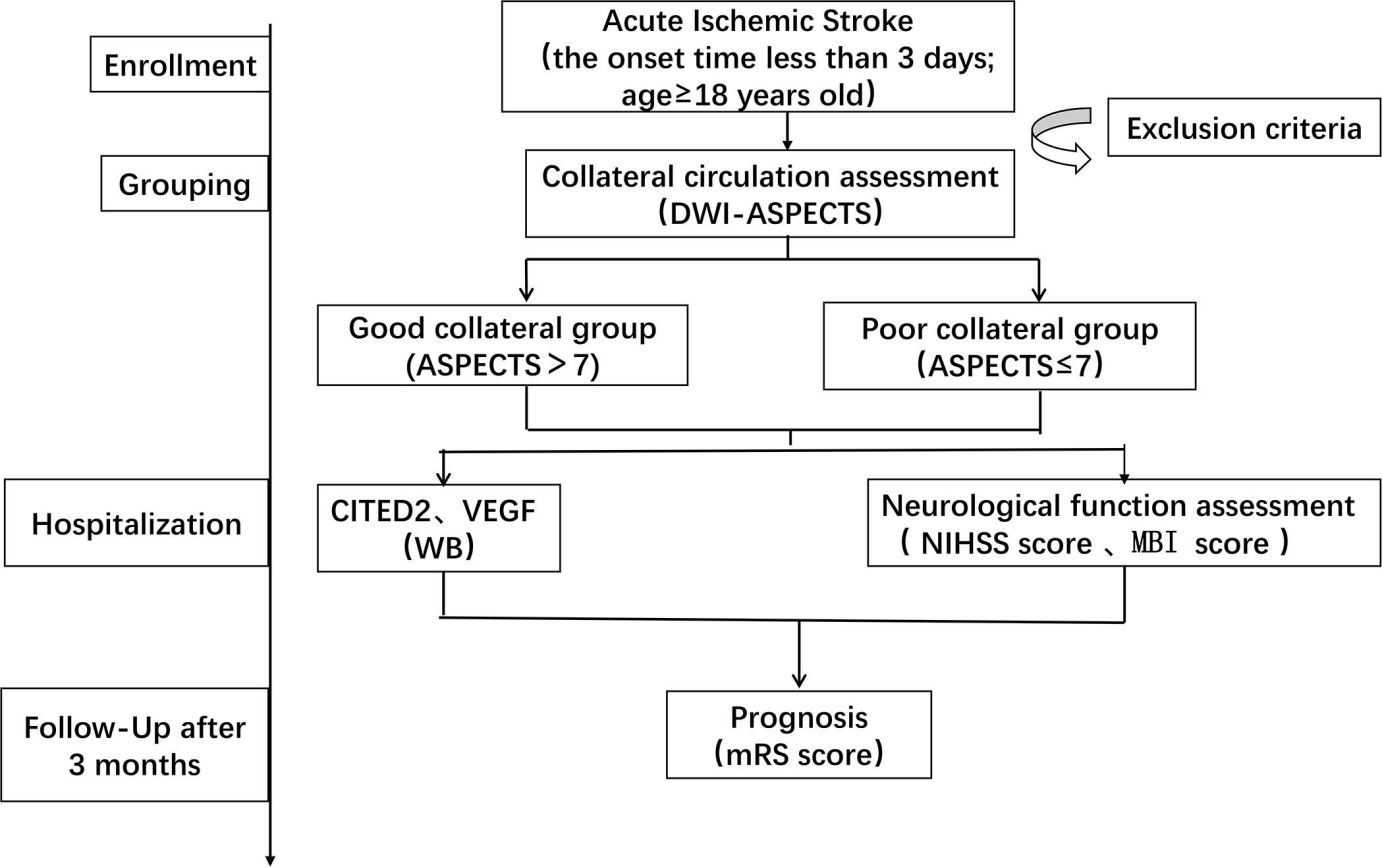
Schedule of enrollment and assessments. DWI-ASPECTS, diffusion weight image–Alberta Stroke Program Early CT Score; CITED2, CBP/P300-interacting transactivator with Glu/Asp-rich C-terminal domain 2; VEGF, vascular endothelial growth factor; WB, Western blot; NIHSS, National Institute of Health Stroke Scale; MBI, Modified Barthel Index; mRS, Modified Rankin Scale.

The institutional ethics committee of the hospital approved this study according to the principles of the Declaration of Helsinki. This was a single-center, prospective, observational study. The patients would perform diffusion-weighted imaging (DWI). Based on the DWI-ASPECTS, we classified patients into two groups: the good collateral group and the poor collateral group. All the patients were collected venous blood samples in the early morning the next day and sent to the laboratory within 1 h. The blood samples had been stored in a 4°C refrigerator before sending to the laboratory. Moreover, the patients were evaluated by the National Institute of Health Stroke Scale (NIHSS) score and the Modified Barthel Index (MBI) score on day 0 and day 7 of hospitalization. Follow-up was carried out after 3 months and recorded clinical outcomes by the Modified Rankin Scale (mRS) score (Figure 1).

### Patient selection

**Figure 2:**
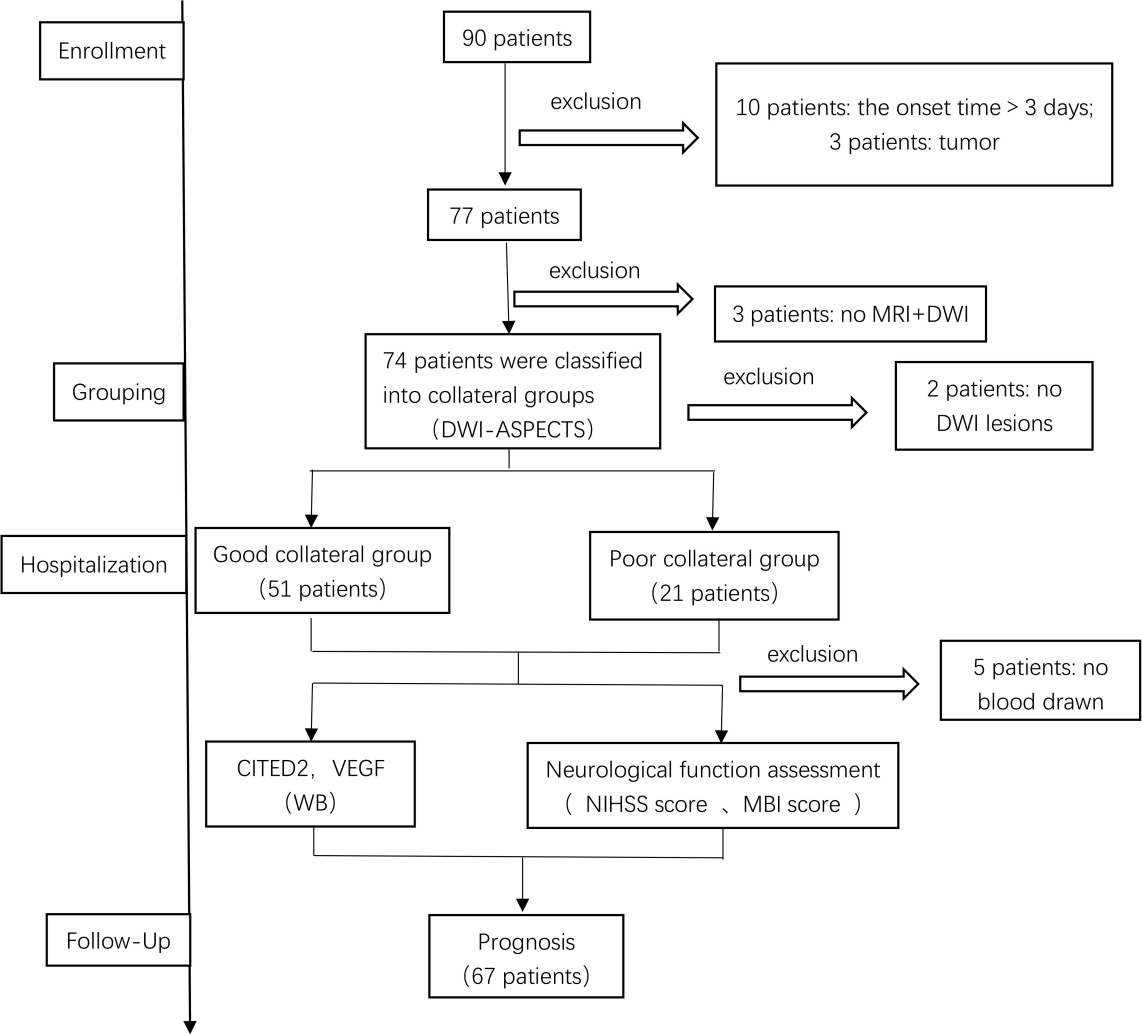
Patient selection. DWI-ASPECTS, diffusion weight image–Alberta Stroke Program Early CT Score; CITED2, CBP/P300-interacting transactivator with Glu/Asp-rich C-terminal domain 2; VEGF, vascular endothelial growth factor; WB, Western blot.

Patients were diagnosed with AIS in the neurology department of our hospital from November 2020 to November 2021. The data of 90 AIS patients were obtained in this study. However, 23 patients were excluded due to various reasons. Of these, 10 patients were excluded due to the onset time>3 days, three patients were excluded because they combined with a tumor, five patients were excluded because they refused to have blood drawn, three patients were excluded because they were not completed the MRI+DWI, and two patients were excluded because the infarct had not been found in DWI. Thus, a total of 67 patients were included in this study (Figure 2).

The inclusion criteria are as follows: ① the onset time <3 days; ② age≥18 years old; and ③ in accordance with the diagnostic criteria in the Guidelines for the Diagnosis and Treatment of Acute Ischemic Stroke in China 2018 (18). The exclusion criteria are as follows: ① the patients had received treatment of thrombolysis or thrombectomy; ② the patients with AIS combined with other diseases that may affect the CITED2 and VEGF expressions, such as acute myocardial infarction, peripheral artery occlusion disease, congenital heart defect, and tumor; ③ the patients exhibited serious heart, liver, or kidney diseases; ④ disturbance of consciousness or mental illness; and ⑤ pregnant and lactating patients.

### Grouping definition

The total score of DWI-ASPECTS is 10 points. The midbrain or pons with high signal was decreased by two points, and other each area was one point. According to infarct lesions involving different circulation, the DWI-ASPECTS corresponding to the circulation was adopted (19, 20). Scores >7 points were defined as the good collateral group, and scores ≤ 7 points were defined as the poor collateral group.

### Peripheral blood mononuclear cell preparation

The 4 ml venous blood samples were collected from all patients within 24 h after hospitalization. The blood samples were diluted with 4 ml of phosphate-buffered saline (Boster, Wuhan) free of calcium and magnesium. Then, 8 ml of venous blood was slowly added to the surface of 8 ml of Lymphocyte Separation Solution (TBD, Tianjin) and centrifuged at 1000 x g for 30 min. The second layer, called the white film lays, was collected and transferred to a centrifuge tube. The 5 ml phosphate-buffered saline free of calcium and magnesium was added to the centrifuge tube and centrifuged at 300 x g for 10 min, and the supernatant was discarded. This step of centrifugation was repeated three times to obtain purified PBMCs.

### Western blot

PBMCs (1 x 10^7^ cells) were lysed by 200 μl RIPA lysis buffer (CWBIO, Beijing) supplement with protease inhibitor cocktail (CWBIO, Beijing) and phosphatase inhibitor cocktail (CWBIO, Beijing). After lysis at 4°C for 15 min, the proteins were centrifuged at 8000 x g for 15 min. The protein concentration in the supernatant of cell extracts was determined using a bicinchoninic acid protein assay kit (Beyotime, Shanghai). Then, proteins were separated by 12% sodium dodecyl sulfate-polyacrylamide gel electrophoresis (Beyotime, Guangdong) and transferred to polyvinylidene difluoride membranes (Millipore, American). The membranes were blocked with 5% no-fat powdered milk (Sangon, Shanghai) in tris-buffered saline (Solarbio, Beijing) with 1% tween (Solarbio, Beijing) for 2 h at room temperature. The membrane was probed with diluted primary antibodies CITED2 (Abclonal, 1:1,000), VEGF (Bioss, 1:1,000), and β-actin (Bioworld, 1:10,000) overnight at 4°C. On the next day, the membrane was re-probed with a secondary antibody, goat anti-rabbit antibody IgG (CWBIO, 1:20,000), labeled by enhanced chemiluminescence hypersensitive luminescent solution (Millipore, American) for 2 h at room temperature, and quantified by densitometry.

### Neurological deficit score

NIHSS was used to quantitatively score the neurological deficit of AIS patients on days 0 and 7 of hospitalization, ranging from 0 to 42 points. The higher the score, the more severe the symptoms.

### Daily life ability score

MBI was used to quantitatively score the daily life ability of AIS patients on day 0 and day 7 of hospitalization, ranging from 0 to 100 points. The higher the score, the better the daily life ability of the patients.

### Prognostic neurological function score

After 3 months, the patient follow-up was carried out by telephone and recorded clinical outcomes of AIS patients by mRS score. A score of 0–2 was defined as a good prognosis of neurological function, and a score of 3–6 was defined as a poor prognosis of neurological function.

### Statistical analysis

Data were analyzed by using SPSS 26.0 software. Quantitative data meeting normal distribution were expressed as the mean ± standard deviation and applied an independent *t*-test in the difference analysis of the two groups. If quantitative data did not conform to the normal distribution, data were expressed as the median (interquartile range) and applied the Mann–Whitney U-test in the difference analysis of the two groups. The counting data were expressed as the cases (percentage) and analyzed by the chi-square test. The correlation between VEGF/CITED2 and collateral circulation was evaluated using binary logistic regression analysis. ROC curve analysis was used to determine the cutoff point, sensitivity, and specificity of VEGF/CITED2. Hypothesis tests were all two-tailed tests, and *P* < 0.05 indicated statistical significance.

## Results

### Baseline characteristics

**Figure 3:**
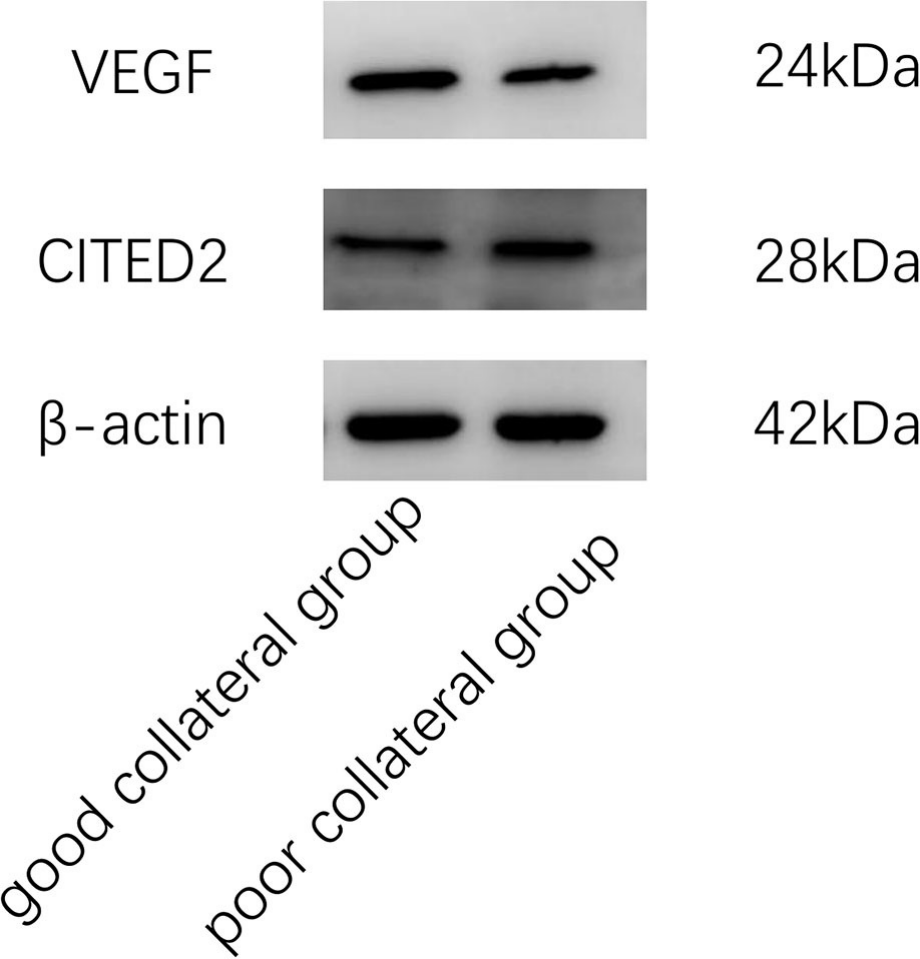
Protein expression of CITED2 and VEGF.

The good collateral group included 46 patients, and the poor collateral group included 21 patients. There was no statistically significant difference between the two groups in general data such as age, gender, hypertension, diabetes, hyperlipidemia, history of coronary heart disease, history of stroke, smoking history, and drinking history (*P* > 0.05) (Table 1). In clinical data, WBC count, neutrophil count, Fib, Hcy, and VEGF/CITED2 were found to be significantly different (Figure 3) (*P* < 0.05). In addition, there was no statistically significant difference between the two groups in other clinical data such as lymphocyte count, neutrophil-to-lymphocyte ratio (NLR), uric acid (UA), total cholesterol (TC), triglycerides (TG), high-density lipoprotein (HDL), low-density lipoprotein (LDL), glycated hemoglobin A1c (HbA1c), CITED2, and VEGF(*P* > 0.05) (Table 2).

**Table 1:** General data of the included patients.

**General data**	**Good collateral group (*N* = 46)**	**Poor collateral group (*N* = 21)**	**t/x^2^**	***P*-value**
Age [mean (SD), y]	64.04 (10.06)	60.38 (13.94)	−1.220	0.227
**Gender [*****n*** **(%)]**			0.082	0.774
Male	29 (63%)	14 (66.7%)		
Female	17 (37%)	7 (33.3%)		
**Hypertension [*****n*** **(%)]**			0.165	0.684
Yes	35 (76.1%)	15 (71.4%)		
No	11 (23.9%)	6 (28.6%)		
**Diabetes [*****n*** **(%)]**			0.295	0.587
Yes	23 (50%)	12 (57.1%)		
No	23 (50%)	9 (42.9%)		
**Hyperlipidemia [*****n*** **(%)]**			0.000	1.000
Yes	7 (15.2%)	3 (14.3%)		
No	39 (84.8%)	18 (85.7%)		
**History of coronary heart disease [*****n*** **(%)]**			-	0.301
Yes	4 (8.7%)	0 (0%)		
No	42 (91.3%)	21 (100%)		
**History of stroke [*****n*** **(%)]**			0.069	0.792
Yes	4 (8.7%)	3 (14.3%)		
No	42 (91.3%)	18 (85.7%)		
**Smoking history [*****n*** **(%)]**			0.165	0.684
Yes	11 (23.9%)	6 (28.6%)		
No	35 (76.1%)	15 (71.4%)		
**Drinking history [*****n*** **(%)]**			-	1.000
Yes	1 (1.5%)	0		
No	45 (97.8%)	21 (100%)		

**Table 2:** Clinical data of the included patients.

**Clinical data**	**Good collateral group**	**Poor collateral group**	**t/x^2^**	***P-*value**
WBC count(×10^9^ cells/L)	8.02 ± 1.93	9.98 ± 3.85	2.217	0.036**
Neutrophil count(×10^9^ cells /L)	5.16 ± 1.95	7.13 ± 3.83	2.234	0.035**
Lymphocyte count(×10^9^ cells /L)	2.03 ± 0.65	2.02 ± 0.94	−0.010	0.992
NLR	2.99 ± 2.21	4.55 ± 3.99	1.674	0.106
UA(μmol/L)	324.48 ± 80.27	339.00 ± 105.40	0.621	0.537
TC(mmol/L)	4.94 ± 1.55	4.85 ± 1.01	−0.241	0.811
TG(mmol/L)	1.88 ± 1.46	1.76 ± 1.10	−0.327	0.745
HDL(mmol/L)	1.03 ± 0.25	1.05 ± 0.22	0.382	0.704
LDL(mmol/L)	3.18 ± 1.41	3.10 ± 0.80	−0.246	0.807
Hcy(μmol/L)	12.28 ± 3.69	14.22 ± 3.50	2.028	0.047**
Fib(g/L)	3.18 ± 0.82	3.75 ± 1.36	2.120	0.038**
HbA1c(%)	7.22 ± 1.92	7.11 ± 1.79	−0.213	0.832
CITED2	1.16 ± 1.77	1.17 ± 0.64	0.034	0.973
VEGF	1.23 ± 0.68	1.02 ± 0.64	−1.182	0.241
VEGF /CITED2	1.41 ± 0.97	0.89 ± 0.26	−2.453	0.017**
**TOAST classification**			3.102	0.078*
Large atheromatous	20 (43.5%)	14 (66.7%)		
**Non- large atheromatous**				
Cardiogenic embolism	0 (0%)	0 (0%)		
Small artery occlusion	24 (52.2%)	4 (19.0%)		
Other defined etiology	2 (4.3%)	3 (14.3%)		
Unknown etiology	0 (0%)	0 (0%)		

WBC, white blood cell; NLR, neutrophil-to-lymphocyte ratio; UA, uric acid; TC, total cholesterol; TG, triglycerides; HDL, high-density lipoprotein; LDL, low-density lipoprotein; Hcy, homocysteine; Fib, fibrinogen; HbA1c, glycated hemoglobin A1c; CITED2, CBP/P300-interacting transactivator with Glu/Asp-rich C-terminal domain 2; VEGF, vascular endothelial growth factor; TOAST, Trial of ORG 10172 in Acute Stroke Treatment.

*P < 0.1; **P < 0.05.

### Clinical prognosis

Compared with day 0, the NIHSS score was decreased and the MBI score was increased in the two groups on day 7. The good collateral group could significantly decrease in NIHSS score and increase in MBI score more than the poor collateral group. The good collateral group has a high percentage (93.5%) of good prognosis and a low percentage (6.5%) of poor prognosis. The poor collateral group has a low percentage (47.6%) of good prognosis and a high percentage (52.4%) of poor prognosis. The differences in clinical prognosis between the two groups were statistically significant (*P* < 0.05) (Table 3).

**Table 3:** Clinical outcome of the included patients.

**Evaluation indicators**	**Good collateral group**	**Poor collateral group**	**t**	***P*-value**
NIHSS score at day 0	5.76 ± 3.09	10.62 ± 7.34	2.918	0.008**
NIHSS score at day 7	2.72 ± 2.21	9.48 ± 9.86	3.107	0.005**
MBI score at day 0	74.83 ± 22.83	50.76 ± 21.80	−4.058	0.000**
MBI score at day 7	87.99 ± 16.39	60.11 ± 25.31	−4.623	0.000**
mRS score at 3 months	0.49 ± 1.13	2.52 ± 2.16	4.067	0.000**
**Prognosis**			15.675	0.000**
Good	43 (93.5%)	10 (47.6%)		
Poor	3 (6.5%)	11 (52.4%)		

NIHSS, National Institute of Health Stroke Scale; MBI, Modified Barthel Index; mRS, Modified Rankin Scale.

**P < 0.05.

### The correlation between VEGF/CITED2 and collateral circulation

Univariate logistic regression analysis was conducted by taking collateral circulation as the dependent variable and age, gender, hypertension, diabetes, hyperlipidemia, history of coronary heart disease, history of stroke, smoking history, drinking history, WBC count, neutrophil count, lymphocyte count, NLR, UA, TC, TG, HDL, LDL, Hcy, Fib, HbA1c, CITED2, VEGF, and VEGF/CITED2 as independent variables. The results demonstrated the WBC count (OR = 0.77, 95%CI 0.63–0.95, *P* = 0.016), neutrophil count (OR = 0.78, 95%CI 0.63–0.95, *P* = 0.016), NLR (OR = 0.83, 95%CI 0.68–1.02, *P* = 0.075), Hcy (OR = 0.87, 95%CI 0.75–1.00, *P* = 0.054), and Fib (OR = 0.59, 95%CI 0.34–1.02, *P* = 0.059) were the risk factors, and VEGF/CITED2 (OR = 190.13, 95%CI 11.04–3,273.83, *P* = 0.000) was the protective factor (Table 4).

**Table 4:** Univariate logistic regression analysis of collateral circulation-related factors in AIS.

			**95% CI**	
	***P*-value**	**OR**	**LCI**	**UCI**
Age(y)	0.226	1.03	0.98	1.08
Gender	0.774	0.85	0.29	2.53
Hypertension(yes/no)	0.685	0.79	0.26	2.52
Diabetes(yes/no)	0.588	1.33	0.47	3.77
Hyperlipidemia(yes/no)	0.921	0.93	0.22	4.01
History of coronary heart disease(yes/no)	0.999	0.00	0.00	-
History of stroke(yes/no)	0.492	1.75	0.36	8.63
Smoking history(yes/no)	0.685	1.27	0.40	4.08
Drinking history(yes/no)	1.000	0.00	0.00	-
WBC count(×10^9^ cells/L)	0.016**	0.77	0.63	0.95
Neutrophil count(×10^9^ cells/L)	0.016**	0.78	0.63	0.95
Lymphocyte count(×10^9^cells/L)	0.992	1.00	0.50	2.01
NLR	0.075*	0.83	0.68	1.02
UA(μmol/L)	0.531	1.00	0.99	1.00
TC(mmol/L)	0.807	1.05	0.72	1.54
TG(mmol/L)	0.741	1.07	0.72	1.60
HDL(mmol/L)	0.699	0.66	0.08	5.56
LDL(mmol/L)	0.803	1.06	0.69	1.63
Hcy(μmol/L)	0.054*	0.87	0.75	1.00
Fib(g/L)	0.059*	0.59	0.34	1.02
HbA1c(%)	0.829	1.03	0.78	1.37
CITED2	0.972	0.99	0.71	1.40
VEGF	0.249	1.82	0.66	4.98
VEGF /CITED2	0.000**	190.13	11.04	3,273.83

WBC, white blood cell; NLR, neutrophil-to-lymphocyte ratio; UA, uric acid; TC, total cholesterol; TG, triglycerides; HDL, high-density lipoprotein; LDL, low-density lipoprotein; Hcy, homocysteine; Fib, fibrinogen; HbA1c, glycated hemoglobin A1c; CITED2, CBP/P300-interacting transactivator with Glu/Asp-rich C-terminal domain 2; VEGF, vascular endothelial growth factor.

*P < 0.1; **P < 0.05.

To adjust confounding factors, variables with *P* < 0.1 (WBC count, neutrophil count, NLR, Hcy, Fib, and VEGF/CITED2) in the univariate logistic regression analysis were tested in further multivariable logistic regression analysis. The results showed that VEGF/CITED2 was an independent protective factor for collateral circulation (Table 5).

**Table 5:** Multivariate logistic regression analysis of collateral circulation-related factors in AIS.

			**95% CI**	
	***P*-value**	**OR**	**LCI**	**UCI**
WBC count (×10^9^ cells/L)	0.855	1.11	0.37	3.34
Neutrophil count (×10^9^ cells/L)	0.806	0.84	0.21	3.34
NLR	0.720	0.91	0.53	1.56
Hcy(μmol/L)	0.219	0.89	0.74	1.07
Fib(g/L)	0.561	0.79	0.36	1.74
VEGF /CITED2	0.001**	165.79	7.25	3,791.54

WBC, white blood cell; NLR, neutrophil-to-lymphocyte ratio; Hcy, homocysteine; Fib, fibrinogen; CITED2, CBP/P300-interacting transactivator with Glu/Asp-rich C-terminal domain 2; VEGF, vascular endothelial growth factor.

*P < 0.1; **P < 0.05.

### The predictive value of VEGF/CITED2

**Figure 4:**
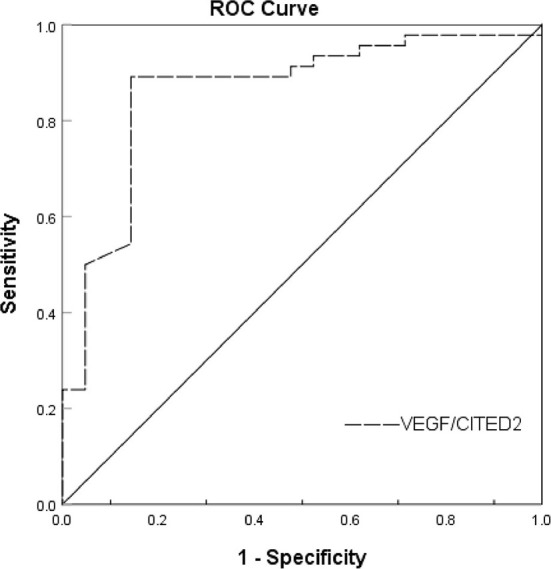
ROC curve analysis of the VEGF/CITED2 in the collateral circulation of AIS.

Receiver operating characteristic (ROC) curve analysis was used to evaluate the predictive value of VEGF/CITED2 in the collateral circulation of AIS. ROC analyses showed an AUC of 0.861 (95%CI 0.761–0.961). The optimal cutoff value of 1.013 for VEGF/CITED2 had a sensitivity of 89.1% and a specificity of 85.7% (Figure 4 and Table 6).

**Table 6:** ROC analysis of the poor collateral circulation in AIS.

	**AUC**	***P*-value**	**95% CI**		**Optimal cutoff value**	**Specificity**	**Sensitivity**
			**LCI**	**UCI**			
VEGF/CITED2	0.861	0.000**	0.761	0.961	1.013	85.7%	89.1%

CITED2, CBP/P300-interacting transactivator with Glu/Asp-rich C-terminal domain 2; VEGF, vascular endothelial growth factor.

**P < 0.05.

## Discussion

By contrasting the good collateral group to the poor collateral group in AIS, we did not only find the good collateral group had a good clinical prognosis, but also that VEGF/CITED2 had a prognostic value for predicting the establishment of collateral circulation in AIS. Moreover, WBC count, neutrophil count, NLR, Hcy, and Fib were the risk factors for collateral circulation and significant differences between the groups.

Collateral circulation was a decisive factor for clinical prognosis in AIS. Our results revealed patients in the good collateral group developed mild neurological deficits, high daily living ability, and good clinical prognosis. These findings had been similar to the result of previous studies (21, 22). This may be explained by the mechanism that good collateral circulation was able to augment brain tissue perfusion and promote the recovery of neurological function, thereby improving the quality of life of patients. It also suggested the DWI-ASPECTS we adopted to evaluate collateral circulation was basically in line with actual collateral status.

VEGF/CITED2 was a protective factor of collateral circulation of AIS. Contrasting with the poor collateral group in clinical data, we found that the VEGF/CITED2 was higher in the good collateral group. Then, we adjusted confounding factors by binary logistic regression analysis and found VEGF/CITED2 was a protective factor of collateral circulation of AIS. However, its correlation related to the collateral circulation of AIS has not been investigated. Previous researches have confirmed that the VEGF and CITED2 are related to collateral circulation. The most widely accepted explanation is that CITED2 plays a negative role in the HIF-1α-VEGF angiogenesis pathway in hypoxia (11, 23, 24). Under hypoxia, CITED2 binds to CBP/P300 competitively with HIF-1α and forms CITED2-CBP/P300 complex. The complex inhibits the expression of VEGF gene encoding protein (25). In addition, it has been proposed that CITED2, as an anti-inflammatory cytokine, may play a role in angiogenesis inhibition through the PPAR pathway (26). The activated PPAR can inhibit the expression of VEGF through the HIF-1α pathway (27).

In general, CITED2 plays a negative role in the VEGF-mediated angiogenesis pathway that has been verified (28–30). VEGF is a potent angiogenic factor. VEGF/CITED2, a comprehensive ratio of VEGF and CITED2, can predict the collateral circulation of AIS more objectively. Indeed, VEGF/CITED2 has not been reported in the literature. Our study implied that VEGF/CITED2 in the good collateral circulation was higher than the poor collateral circulation, which was a statistically significant difference. We speculated that VEGF/CITED2 may be a balance point in the collateral circulation. When good collateral circulation is established, the inhibition of CITED2 is weaker and VEGF mainly plays a pro-angiogenic effect. Conversely, when poor collateral circulation is established, VEGF plays a weaker pro-angiogenic effect and CITED2 mainly exerts an effect in negative regulation. On the contrary, VEGF and CITED2 are expressed in mononuclear macrophages (13, 31). After AIS, the brain parenchyma generates the cytokines and chemokines leading to inflammatory cells in peripheral blood being attracted to infiltrate the ischemic area (32, 33). Thus, we detected VEGF/CITED2 of PBMCs by Western blot. Our results showed significant differences in the VEGF/CITED2 of PBMCs between these two groups.

Further analysis of the ROC curve results showed that the VEGF/CITED2 of PBMCs was an independent predictor of AIS. The optimal cutoff value of 1.013 means the AIS patients are more likely to have good collateral circulation if VEGF/CITED2>1.013. At this point, the VEGF/CITED2 had a sensitivity of 89.1% and a specificity of 85.7%. Due to the lack of studies on the correlation between VEGF/CITED2 and cerebral collateral circulation, it was difficult to make a direct comparison in the optimal cutoff value of VEGF/CITED2. At the same time, we looked through the literature on biomarkers of cerebral collateral circulation. The sphinganine-1- phosphate (S1P) is a bioactive lipid that acts on receptors to mediate various cellular processes, including cell growth, differentiation, angiogenesis, and immunoregulation (34, 35). Recently, Fang Yu et al. classified AIS patients into two groups (the good collateral group and the poor collateral group) by the Tan score of CTA. They tested S1P in plasma and found that the AUC of S1P was 0.738 (95%CI 0.60–0.85) (36). They considered that the S1P was an independent predictor of cerebral collateral circulation. Because of different study populations and collateral circulation grouping, the comparison could not be performed for S1P and VEGF/CITED2. Thus, the biomarker of cerebral collateral circulation has been poorly studied, which still needs to be further explored in future.

Besides, it was found that the differences between the good collateral group and the poor collateral group were significant in AIS such as WBC count, neutrophil count, NLR, Fib, and Hcy. In univariate logistic regression analysis, they also were the risk factors for collateral circulation. There are the following mechanisms that may be involved: After AIS, the WBC is attracted to infiltrate the ischemic area. Among these, neutrophils are the first immune cells to the ischemic tissue and further aggravate ischemic damage (37). A few reports have shown that neutrophils inhibit angiogenesis by releasing elastase and α-defensins (also known as human neutrophil peptides) (27, 38). The leukocyte has been considered to be a marker of inflammatory response after stroke and NLR is an objective indicator of neutrophil and lymphocyte. Thus, the high levels of the inflammatory response are deleterious to collateral circulation. Hcy can cause damage to vascular endothelial cells, leading to vascular endothelial cell dysfunction and inhibition of collateral circulation (39, 40). The vascular endothelial cells are required for the localized degradation of the fibrin matrix so that they can proliferate, migrate, and form capillaries. A high level of Fib can inhibit this process, which in turn inhibits collateral circulation (41–43). However, when in the multivariate logistic regression analysis, WBC count, neutrophil count, Fib, and Hcy were found no statistically significant (*P*>0.05). We speculate that there are two possibilities. First, the small sample size may lead to false-negative results. Second, there may exist an association among these factors such as WBC count, neutrophil count, NLR, and VEGF/CITED2. The VEGF and CITED2, secreted by inflammatory cells, play an anti-inflammatory effect (13, 44). When the VEGF/CITED2 was controlled by multivariate logistic regression analysis, the statistical correlation of WBC count, neutrophil count, and NLR disappears.

In this study, we were pleasantly surprised that VEGF/CITED2 is related to the collateral circulation of AIS. As a predictor for collateral circulation, VEGF/CITED2 is also an independent protective factor. However, there are some limitations to this study. For one thing, we could not further adjust confounders due to the small sample size. For another, the DWI-ASPECTS might have certain effects on patients' grouping. Although this score was performed for an initial evaluation of clinical prognosis, further evaluation of the predictive value of VEGF/CITED2 is still needed by adopting DSA or other precise methods.

## Conclusion

Collateral circulation was a decisive factor for clinical prognosis in AIS. Moreover, VEGF/CITED2 in PBMCs had been associated with the collateral circulation of AIS. It was an independent protective factor and has a potential predictive value in the collateral circulation of AIS.

## Data availability statement

The original contributions presented in the study are included in the article/supplementary material, further inquiries can be directed to the corresponding author/s.

## Ethics statement

The studies involving human participants were reviewed and approved by the Institutional Ethics Committee of the Zhujiang Hospital. The patients/participants provided their written informed consent to participate in this study.

## Author contributions

ML: investigation, methodology, writing—original draft, and writing—reviewing and editing. YL and ZX: investigation, methodology, and writing—original draft. XY: data analysis and investigation. JC: investigation and supervision. ST: data analysis and supervision. PZ: conceptualization, resources, and writing—reviewing and editing. YG: conceptualization, resources, writing—reviewing and editing, supervision, and project administration. All authors read and approved the final manuscript.

## Funding

This work was supported by the Dean Fund of Zhujiang Hospital, Southern Medical University (No. yzjj2020qn02 to YG).

## Conflict of interest

The authors declare that the research was conducted in the absence of any commercial or financial relationships that could be construed as a potential conflict of interest.

## Publisher's note

All claims expressed in this article are solely those of the authors and do not necessarily represent those of their affiliated organizations, or those of the publisher, the editors and the reviewers. Any product that may be evaluated in this article, or claim that may be made by its manufacturer, is not guaranteed or endorsed by the publisher.
